# Use of aripiprazole in dysthymic disorders. Purposely a case

**DOI:** 10.1192/j.eurpsy.2023.1778

**Published:** 2023-07-19

**Authors:** N. Ogando Portilla, S. M. Bañon Gonzalez, M. Agudo Urbanos, M. Martinez Cortes, O. Sobrino Cabra

**Affiliations:** 1 Psychiatry, HOSPITAL INFANTA SOFIA; 2 Psychiatry, HOSPITAL LA PRINCESA, madrid; 3 Psychiatry, Hospital General de Alicante, Alicante; 4 Psychiatry, Hospital Infanta Elena, madrid, Spain

## Abstract

**Introduction:**

Dysthymia is a chronic mood disorder with similar but less severe features than major depressive disorder. Compared to the latter, major depressive episodes of dysthymic disorder are more spaced, less intense, and more persistent.

The most effective treatment is usually the combination of serotonin reuptake inhibitor antidepressant drugs with behavioral, cognitive, interpersonal and group psychotherapies.

The reality is that there are few clearly effective treatments to treat this disorder which makes the symptoms even more chronic which has a negative impact on the functionality of patients with clear influence at a personal and work level. Without treatment, dysthymia sometimes progresses to major depression, called “double depression” what can be a most serious problem.

**Objectives:**

Finding new lines of treatment or management in these patients seems to be essential because of the inability that can occur in some of them and the high demand they can produce.

**Methods:**

A 45-year-old woman diagnosed of dysthymia has been followed for more than 10 years. Multiple visits to the emergency room and several outpatient mental health services. absenteeism and great repercussion in the family environment. Many side effects to antidepressants and a benzodiazepine overuse tendency. She has been receiving psychotherapeutic treatment for many years with little effectiveness. Worsening of the symptoms with the appearance of obsessiveness around what is happening to her

**Results:**

Several alternative treatments are tested for the management of anxious depressive and obsessive symptoms being Aripiprazole 10mg the only effective one with almost complete recovery of symptoms. The patient returns to work and significantly improves her family situation.

**Conclusions:**

Dysthyma is a disorder with difficult pharmacological and psychological management. Trying different little-used treatments can open up a different view about the disorder.

The use of serotonin reuptake inhibitor antidepressant drugs is not always effective and the risk posed by using benzodiazepines for long time forces us to look for other treatments for the control of the main symptoms The use of aripiprazole at moderate doses may be a good new way to control symptoms.

**Disclosure of Interest:**

None Declared

